# Anatomical Description of the Parts Concerned in Inguinal and Femoral Hernia

**Published:** 1835-04-01

**Authors:** 


### ANATOMICAL DESCRIPTION OF THE PARTS CONCERNED IN INGUINAL AND FEMORAL HERNIA, TRANSLATED FROM THE FRENCH OF *M. Jules Cloquet;* with Lithographic Plates from the original Etchings, and a few additional explanatory Notes. By *Andrew Melville M'Whinnie,* Assistant Teacher of Practical Anatomy at St. Bartholomew's Hospital. Octavo, pp. 50; 4 Plates. London, 1835.

WE are not in the habit of noticing at any length works exclusively devoted to anatomy. The majority of the readers of a periodical publication can scarcely be supposed to feel a strong interest in those recondite anatomical researches, which are properly appreciated, and indeed relished only by a few. Yet the character of the student and that of the practitioner are so inseparably mingled in the present day, that the line of distinction between them is vague, perhaps altogether undistinguishable. We mean that the period of study is necessarily spread over the whole of a professional existence, that none can stop and exclaim "ἑυρηκα," "the goal of our researches is attained." Every day discloses new facts or new deductions—

things which were unknown become familiar, and things which were familiar are clothed in new relations. It is the aim and object of periodical literature to diffuse this rising knowledge—to irrigate the profession with the fertilizing waters.

We are tempted to notice, and with some degree of circumstantiality, the present translation of the work of M. Cloquet, because a great deal has been done of late years in determining the anatomy of the parts concerned in hernia; and because a summary of what is actually ascertained, must be highly useful to those whose situation or whose means preclude the possibility of obtaining or of studying the many separate works or papers on the subject. Some surgeons regard the anatomical investigations of hernia with horror, others with contempt. The former are dismayed by the numerous parts with innumerable names—the latter consider the precision of the schools inapplicable or valueless in practice. We will not moot the question with either of these gentlemen; but indulge in the mild and not indefensible dogmatic assertion, that an exact acquaintance with the structure of parts in which many delicate operations are performed, can never be injurious, and must often be of service to the operator. We say exact acquaintance, for that loose kind of information which evades the difficulties it is too indolent to conquer, betrays rather than supports those who place their trust in it.

The translator of the present work observes of the original, that the estimation in which it is held by the most experienced anatomical teachers, affords his apology and his reason for presenting it to students in this country. For the same reason, and for that on which we have already been discursive, we shall place its substance before our readers. The surgeon in the provinces and in the colonies may con the laborious anatomical lesson with profit, and indeed with pleasure.

The parts concerned in inguinal hernia are described first, and those connected with femoral hernia are noticed last.

### Inguinal Hernia.

The parts described are successively these :—the aponeurosis of the external oblique muscle—the fascia superficialis—the obliquus internus muscle—the cremaster—the transversalis muscle—the rectus—the pyramidalis—the fascia transversalis—the epigastric vessels—the inguinal canal—the spermatic cord—the peritoneum. Such are the parts we are now to pass in review. On some we shall dwell fully, on others we shall lightly touch. It would be idle to repeat descriptions contained in all anatomical works, and we therefore select for notice the matter which is less accessible, and less familiar. We must crave permission to indulge in quotation, for descriptive anatomy is not easily condensed. Reversing the order adopted by M. Cloquet, we shall consider the fascia superficialis previously to the aponeurosis of the external oblique.

### *Of the Fascia Superficialis.*

The aponeurosis of the external oblique is covered by this fascia, which intervenes between it and the skin. The following are the results of careful dissections on the part of M. Cloquet.

"First being simply formed by a whitish and condensed cellular tissue, it covers the abdominal muscles and aponeuroses; it adheres but slightly to the latter, but it is so intimately connected with the muscles as to render their dissection difficult. Internally, it is continuous with that of the opposite side, by passing in front of the linea alba, from which it is separated with facility; externally, it passes to the crista of the ilium, becomes entirely cellular, and then covers the glutæus maximus and medius; in front of the abdomen it is difficult to ascertain the precise direction of its fibres; it contains the subcutaneous vessels of the abdominal parietes; inferiorly it passes in front of Poupart's ligament, to the external part of which it is rather strongly adherent. Surrounding the external abdominal ring without being firmly connected with it, the fascia superficialis extends upon the spermatic cord, to which it gives, as abovementioned, a thin cellular sheath, easily separated, and which accompanies it to the bottom of the scrotum. This sheath also embraces the tunica vaginalis and the testicle, and identifies itself lastly with a white and triangular fasciculus of fibres, which connect the latter organ to the scrotum and ramus of the ischium, (they are the remains of that structure which J. Hunter called the gubernaculum testis.) In many individuals this sheath is so thin that the fibres of the cremaster as well as the vessels of the cord which it surrounds may be seen through it. Two or three superficial arteries derived from the femoral, pass transversely, some in front of, others behind the cord to the root of the penis: they are contained in the substance of the fascia now described, and are accompanied by veins, which terminate in the femoral vein, through the opening in the fascia lata which gives passage to the vena saphena. On the inner side of the external ring, the fascia superficialis extends to the root of the penis, and is continuous with the loose cellular tissue by which the penis is surrounded.

Below the crural arch, the fibres of this fascia are very distinct; they are parallel to the bend of the thigh, and form meshes of considerable size, irregularly disposed, leaving spaces filled with fat or lymphatic glands. Externally it reaches the outer part of the thigh, and rests upon the surface of the fascia lata, which it also covers internally, and where it is fixed above to the ramus of the ischium near to the root of the corpus cavernosum.

The fascia superficialis passing in front of the aponeurotic opening for the vena saphena, adheres more or less intimately to its edge, and afterwards descends upon this vein, by which it is separated from the fascia lata." 4.

In fat subjects the fascia is thin and indistinct; in thin subjects it is thicker, whiter, and stronger.

### *Of the Aponeurosis of the External Oblique.*

Under this head we need only notice the manner in which the external abdominal ring is produced. The fibres of the aponeurosis of the external oblique separate near the pubes into two fasciculi, which are known by the name of columns of the inguinal ring. Of these columns, one is internal and superior; it is broad and flattened, and is fixed to the front part of the symphisis pubis, its fibres crossing those of the opposite side.* The other is external and inferior; it is of a rounded form, and much stronger than the internal column—it is attached to the spine of the pubes, and there is an extension of it to the crista of the pubes.† Between these two aponeu-

* These decussating fibres are continuous with the suspensory ligament of the penis.—*Eds.*

† This is described subsequently; it is the ligament of Gimbernat.—*Eds.*

rotic columns there is an aperture generally triangular, but varying considerably in its form and dimensions:—the ring of the external oblique or the inguinal ring. Its base* is formed by the pubes; its sides by the columns; its summit, situated superiorly and externally, is the point where the fibres of the aponeurosis of the external oblique separate into the two fasciculi. The summit is rounded in consequence of superficial aponeurotic fibres taking a transverse direction, and uniting the two columns which they cross at an angle more or less acute.

These transverse fibres arise from the lower part of the crural arch, or the ligament of Poupart, where they are closely collected together; from thence they proceed in a radiating manner obliquely upwards and inwards towards the linea alba; a portion of them in front of the inguinal ring, which they narrow, and then become lost in the aponeurosis of the external oblique. In some individuals they are strongly developed, whilst in others they are very thin, or can scarcely be said to exist. In the female they are much weaker than in the male; they prevent the separation of the columns, and strengthen the lower part of the aponeurosis, which, indeed, is of greater thickness in this situation than elsewhere.

The great diameter of the inguinal ring is parallel to Poupart's ligament; so that the summit is towards the anterior superior spine of the ilium, whilst its base is towards the pubes. The circumference of the ring gives origin to a very thin cellular expansion,† which surrounds the cremaster muscle, and is soon lost upon the spermatic cord, by being confounded with the cellular covering, which the latter receives from the fascia superficialis; but from which it was at first quite distinct.‡

*Of the Obliquus Internus Abdominis.*

We are not under the necessity of dwelling on the ordinary anatomical relations of the internal oblique muscle. The manner in which its aponeurosis divides, in order to constitute the sheath of the rectus, is of course familiar. But the sheath of the pyramidalis is not usually described. According to our author, it is formed anteriorly by the united aponeuroses of the external and internal oblique, and posteriorly by the layer derived from the transversalis. The anatomy of the lower border of the internal oblique

---

* The base is also known as the internal angle of the ring; and the summit as the external angle.

† "This cellular expansion constitutes the inter-columnar fascia of some authors; it is also represented as becoming the tunica aponeurotica of inguinal hernia."—*Translator*.

‡ "The inguinal ring is of less extent, and its columns are not so strong in the female as in the male. Sometimes it consists of a very small rounded aperture, closely embracing the spermatic cord or the round ligament; in other instances it has a very elongated form, and the spermatic cord passes out through its external angle and over the inferior column, at some distance from the pubes. In several subjects I have found the columns joining each other at a distance not exceeding one or two inches from the anterior superior spine of the ilium; the ring in such instances was of considerable size, and the oblique fibres which crossed it gave it a square form."—*Cloquet*.

and transversalis, a subject on which some doubt has been experienced and some difficulty exists, is thus given by M. Cloquet.

" The lower border of the transversalis, composed of very thin pale fibres, passes in a transverse direction above the spermatic cord, at the point where it enters the inguinal canal, that is, on a level with the superior opening of this canal internally, it is inserted into the lower part of the linea alba, and slightly into the pubes, by uniting with the aponeurosis of the internal oblique. The inferior edge of the latter, attached, as I have stated, to the crural arch, descends parallel to it, covering the spermatic cord in the inguinal canal, and is fixed inwardly to the pubes. It passes over the cord, just at the point where the latter escapes from the inferior opening of the inguinal canal. The fibres of the internal oblique there change their direction, to give origin to the cremaster—those fibres which were straight and nearly horizontal, become curved and vertical. They pass through the ring, and then, descending below it, form, in front of the cord, loops or arches, with their concavities directed upwards, and which may be traced to the bottom of the scrotum. The fibres are applied upon the whole of the anterior surface of the tunica vaginalis, and of the spermatic cord. These arches are of greater extent, inferiorly: occasionally one of them, single at its extremities, separates into two, towards its middle, and encloses a sort of crescentic space. They are all united, towards the external ring, into two triangular fasciculi. The one external, and the stronger, passes through the corresponding part of the aperture; the other, internal, and less developed, enters the ring, behind the inner column, to be attached to the pubes. This disposition, which is constant, has been but imperfectly understood by anatomists. The greater number have only described the external fasciculus of the cremaster; others have spoken only in a vague manner of the fibres which are inserted into the pubes. No anatomist, to my knowledge, has described those muscular arches which I have shown to exist in front of the spermatic cord; I therefore think it useful to treat this part of the anatomy in detail." 6.

The arches of the cremaster are with difficulty distinguished in front of the testicle and tunica vaginalis, and usually the muscle is insensibly lost upon the lower part of the proper sheath of the spermatic cord. In many subjects the arches of the cremaster are found behind the cord, as well as in front of it; they are much less distinct than the anterior.

M. Cloquet feels assured, from many dissections, that the cremaster does not exist antecedently to the period at which the descent of the testicle takes place. It arises from the testis sometimes passing under, sometimes through the inferior fibres of the internal oblique. The hernial sac, in descending, acts in a manner similar to the gubernaculum testis, and augments the number of the fibres of the cremaster at the expense of those of the internal oblique, which it draws down with it through the inguinal ring. From the consideration of these circumstances it must be evident, that the testis, as well as the sac of an external inguinal hernia, when it exists, are sustained on every side by the cremaster, and not merely on the outer, as the common descriptions of the muscle would imply. The external triangular fasciculus of the cremaster is usually stronger than the inner, or pubic. Occasionally the internal fasciculus appears deficient, in consequence of the length of its aponeurotic fibres; sometimes it would seem to be actually absent.

" In the female, the lower fibres of the internal oblique are much thinner than in the male; they pass above the round ligament without entering the inguinal ring: in the natural and healthy state, therefore, we find no trace of cremaster.*

The cremaster muscle is covered by the prolongation of the fascia superficialis, which passes downwards upon the spermatic cord, and more immediately by an expansion of very fine cellular tissue, which extends from the circumference of the external ring, and which becomes intimately connected with the cremaster; it is also applied upon the proper sheath of the cord,† to which, inferiorly, it becomes closely connected. Superiorly, we may in most cases separate these two coverings from each other.

The inferior border of the internal oblique, is, in many subjects, so confounded with the transversalis, that we cannot be certain that the cremaster does not receive fibres from the latter muscle." 12.

### *Of the Transversalis Muscle.*

On this head we find nothing deserving of attention.

Neither will the rectus nor pyramidalis detain us.

### *Of the Fascia Transversalis.*

Of this fascia we shall only advert to the portion which affords an investment to the cord and to a hernia. What is termed the superior opening of the inguinal canal, or the inner ring, is an elongated aperture, the great diameter of which is vertical, presented by the fascia transversalis, above and towards the middle of Poupart's ligament. Its internal edge, thicker and more strongly marked than its external, is sustained by falciform fibres, detached from the arch itself. This orifice must not be considered as a simple foramen, but rather as the wide entrance to a funnel-shaped canal, which, in the male, receives the vessels of the spermatic cord, and of which it constitutes the sheath by extending over them. In the female, it gives passage to the round ligament of the uterus; it is of much smaller dimensions than in the male, and is sometimes with difficulty recognised. The sheath which it forms around the cord consists of a long cellular canal, easily separable from it, descending with the spermatic vessels through the inguinal canal, and accompanying them to the upper border of the testis, where it becomes lost in the cellular tissue which surrounds the tunica vaginalis. The prolongation in question of the fascia has received, as the translator observes, the various names of fascia canalis, fascia infundibuliformis, and tunica vaginalis of the cord. It has been described by some authors as constituting the fascia propria to the hernial sac.

Sometimes this cellular sheath of the cord, derived from the fascia trans-

---

* "In the female, the sac of an external inguinal hernia, in its descent, frequently carries with it the fibres of the internal oblique and forms an accidental cremaster extended in front of the tumor, and the festoons of which, very pale, separated from each other, and scarcely visible from their thinness, unite into two triangular fasciculi at each angle of the ring, as in the male. A very careful dissection is required to discover these fibres, which are not equally developed in all cases. Often I have not succeeded in finding them."

† "This sheath originating from the fascia transversalis, is a prolongation of the sort of infundibulum which the latter presents, to form the superior opening of the inguinal canal."

versalis is distinguishable from the laminated cellular tissue which unites the spermatic vessels, and which is derived from the external surface of the peritoneum ; sometimes their separation is impracticable.

The epigastric artery passes between the fascia transversalis in front and the peritoneum behind.

The following is M. Cloquet's summary of the anatomy of the fascia transversalis.

"The fascia transversalis then, is an aponeurosis, which varying* in thickness, arises from the posterior edge of Poupart's ligament, from the fascia iliaca, from the external edge of the tendon of the rectus muscle, and is continuous above with the cellular tissue situated upon the internal surface of the abdominal muscles; inferiorly and towards the middle of the crural arch it forms a membranous canal, which commences by a wide opening, directed posteriorly and externally, and of which the internal edge is much the stronger—this canal descends around the spermatic vessels to compose their proper sheath. The fascia transversalis supports the peritoneum behind, and is separated from it by the epigastric artery ;† in front it corresponds with the transversalis muscle, and with the aponeurosis of which it is often so intimately connected, that it can only be distinguished from it by the different direction of its fibres." 17.

*Of the Epigastric Vessels.*

We will not speak of the origin of the epigastric artery, nor of its usual course. The ordinary works upon anatomy are sufficiently explicit on these points. Yet there are three passages in the description of M. Cloquet to which we may usefully direct attention.

1. The relations of the artery to the inner ring are interesting and important. In general, says M. Cloquet, the epigastric artery runs immediately upon the internal border of the superior aperture of the inguinal canal, so that the spermatic vessels on entering this canal, appear at first sight to wind round this artery, but which in reality supports them only in a very trifling degree. If we remove this vessel, we find in front of it the internal border of the opening in the fascia transversalis, which, in fact, is the part which sustains the cord, and which prevents its being carried inwards. In some subjects the epigastric artery is situated at the distance of four or five lines to the inner side of this opening, and is not at all in contact with the spermatic vessels at the point where they form a curve to enter the inguinal canal. The situation of the umbilical artery varies considerably. Converted into a fibrous cord, it is in some instances situated immediately on the inner side of the superior opening of the inguinal canal; in others, it is at some distance from it. We may conclude therefore, first, that the spermatic vessels are always sustained internally by the inner border of the opening in the fascia transversalis; secondly, that in most instances the epigastric

---

* "The fascia transversalis is in some individuals extremely thin ; in others, towards the rectus muscle, it is composed of very strong bundles of fibres, so disposed that open spaces, varying in form and number, are left between them."

† "The fascia transversalis possesses the greatest strength between the superior opening of the inguinal canal and the rectus muscle. In this situation it is opposite to the posterior part of the inguinal ring, from which it is separated merely by the thin fibres of the internal oblique and transversalis, which are attached to the pubes."

artery contributes to their support; thirdly, that in some cases the umbilical artery assists these two parts in maintaining the cord in its situation.

2. The relations to the outer ring should not be disregarded. Under ordinary circumstances the epigastric artery is placed at the distance of about an inch from the outer side of the external ring. But M. Cloquet notices the following exceptions to this, the usual location of the vessel.

When, however, the inguinal ring is of considerable length, its external angle, or rather its summit, is situate only a few lines distant from the epigastric artery. I will here observe, that the proximity of the external angle of the ring to the epigastric artery is consequent on, 1st, the deviation of the artery from its proper course, as caused by an external inguinal hernia inclosed within the canal; 2ndly, the elongation of this angle towards the epigastric artery, which retains its proper situation as from an internal inguinal hernia; 3dly, these two parts, in some cases appearing to become opposite each other, and which we frequently observe in large external inguinal herniæ, where the obliquity of the inguinal canal is destroyed.

3. The epigastric artery is well known to be situated on the outer side of the neck of the sac of a direct, ventro-inguinal, or, as M. Cloquet terms it, internal inguinal hernia. Our author's observations on this subject are of the following specific character. Before the artery reaches the rectus muscle, it forms the external boundary of a triangular space, the base of which is formed by Poupart's ligament, and the internal border by the rectus muscle. The extent of this space is proportionate to the distance at which the epigastric artery is placed from the symphysis pubis. Internal inguinal herniæ occur in the lower part of this triangular space, most frequently very near to the tendon of the rectus; it very rarely happens that the hernia is found on the outer side of the space, that is, near to the epigastric vessels.

We proceed, after considering the component parts of the inguinal canal, to examine the canal itself.

### *Of the Inguinal Canal.*

We will not pursue M. Cloquet through his accurate review of the component parts and the general characters of the inguinal canal. We will simply pause at one or two unconnected points.

1. It has been stated that the aponeurotic fibres of the external oblique, where they form the *internal* pillar of the outer ring, decussate with those of the corresponding muscle on the other side of the abdomen, and become continuous with the suspensory ligament of the penis. But it should also be observed that the *external* pillar of the outer ring is brought into relation with the linea alba, and in this manner:—Radiating aponeurotic fibres ascending from the pillar in a diverging manner, to be attached to the lower part of the linea alba, after having passed behind the internal column of the ring. These fibres have received the name of the triangular fascia or ligament. Thus the internal pillars of the ring on either side decussate; and the external pillars are connected at the linea alba, by means of this triangular aponeurosis.

2. The external ring, or external opening of the inguinal canal presents much variety in the extent, form, and strength of the aponeurotic fibres which constitute its boundaries, and which must influence the dimensions of the inguinal canal, the amount of resistance offered to the viscera in their protrusion, and the degree of strangulation which they may suffer. M. Cloquet appends a note expressing the result of his observations upon this point, observations which are not unimportant.

" I have already noticed many of the varieties in the formation of the inguinal ring; here I will observe that the extent of the inguinal canal bears an inverse ratio to that of its inferior aperture. In some subjects, the ring extending to the centre of the crural arch, the cord, as it passes out, is placed at some distance from the pubes; the inguinal canal, then, having a direction from behind forwards, is very short, and the testicle can easily re-enter it. Frequently the fascia superficialis is united so loosely to the parts adjacent to the inguinal ring, that when the testicle is pushed upwards, it may become placed between that fascia and the aponeurosis of the external oblique. In examining such a case, we might be led to believe, that the testis has introduced itself into the inguinal canal; but if the gland be pushed downwards again, we do not discover the large opening, which permits the easy introduction of the finger, and which is formed by the dilated inguinal ring in the few individuals in whom the testes can really re-enter the canal." 21.

3. The differences observed in the form and the dimensions of the inguinal canal in the male and in the female are not undeserving of attention.

The canal is wider, and its apertures are much more distinct in the male than in the female. Its direction, which usually corresponds with that of the crural arch, is also a little more oblique in the former than in the latter.* The occurrence of inguinal hernia is influenced by the differences in the dimensions of the inguinal canal, and which depend on the age and sex.

The following are the admeasurements offered by M. Cloquet. We may premise two remarks: 1, that it is rare to find two individuals in which the measurements exactly correspond; 2, that no obvious explanation can be found in the physical conformation, for the greater prevalence of herniæ on the right side than the left. To return to M. Cloquet's measurements.

| | MALE. *Inches.* | FEMALE. *Inches.* |
|---|---|---|
| " From the symphysis pubis | | |
| to the anterior superior spine of the ilium .. | 5¾ | 6 |
| to the tuberosity of the pubes ............ | 1⅛ | 1⅜ |
| in the inner margin of the external abdominal ring ......................... | 0⅞ | 1 |
| to the inner margin of the superior aperture of the inguinal canal ................ | 3 | 3¼ |
| to the middle of the iliac artery .......... | 3⅛ | 3⅜ |
| to the middle of the iliac vein ............ | 2⅝ | 2¾ |
| to the origin of the epigastric artery ...... | 3 | 3¼ |
| to the point where the epigastric artery passes on the inner side of the superior opening of the inguinal canal ............ | 2¾ | 2⅞" 22. |

" * If a horizontal line be drawn on a level with the pubes, and an oblique one from the symphysis pubis to the anterior superior spine of the ilium, it will be found that in the female, these two lines will meet at a more acute angle than in the male, and which depends on the less degree of elevation and the greater breadth of the pelvis in the former: in the female, also, the crural arch is more horizontal. This difference, however, is not striking in many individuals."

The spermatic cord and the peritoneum, which form the two concluding subjects discussed by M. Cloquet in reference to inguinal hernia, present only one anatomical feature to arrest us. It is this. Towards the cavity of the abdomen, the point at which the spermatic vessels pass into the canal is marked by a conical depression of the peritoneum, which has the form of a little funnel, and frequently sends a prolongation in front of the cord. In the majority of subjects, however, the peritoneum passes over the superior aperture of the inguinal canal without being extended into its interior. M. Cloquet has directed much attention to this depression in the peritoneum, a depression of some importance in connexion with hernia, and congenital hydrocele.

" This little depression (says he) is occasionally situated at the distance of five or six lines to the outer side of the opening in the fascia transversalis. In many subjects I have discovered it lying in front of the spermatic vessels in the iliac fossa to which it had probably been drawn. In most instances this depression does not correspond with the apex of the pyramid formed by the larger of the two fossæ, which extends more internally. Occasionally it is continuous with a cellular filament, which consists of the remains of the tunica vaginalis, or more properly of its canal of communication with the peritoneum in the fœtus. I have met with the remains of the tunica vaginalis in male subjects of all ages ; and it is a singular circumstance that they should be nearly as frequently found in the old as in the young subject, The following are the principal varieties which have occurred to my observation.

1st. The little depression of the peritoneum adheres simply to the spermatic cord, in front of which it is always situated, through the medium of a dense cellular tissue which extends upon the cord in the form of a whitish filament, which becomes thinner and thinner, and is soon lost in the cellular membrane which unites the spermatic vessels.

2dly. The depression of the peritoneum is continuous with a long whitish cord, of a fibro-cellular texture, which may be traced as far as the tunica vaginalis.

3dly. This cord, instead of being solid throughout its whole extent, presents at different points oblong sacculi, two, three, or four in number, separated from each other by contracted portions. These cavities usually communicate with each other by very narrow orifices, and will admit of inflation.

4thly. There exists often only one oblong cavity, of an inch or an inch and a half in length, either entirely contained within the inguinal canal, or projecting from it in a trifling degree, large at its fundus, and continuous with the peritoneum by a narrow neck, or sort of pedicle, which sometimes contains a small canal by which the cavity may be inflated, but which at other times is solid. In this case, the cavity does not communicate with the peritoneum, and represents a kind of cyst, which might be mistaken for the remains of a hernial sac. At the point where the pedicle is attached, the peritoneum presents a small cicatrix, more or less marked. The parieties of this cyst, and of the others just mentioned, are more or less thin, transparent, and elastic; occasionally they are white, opaque, and easily torn ; their interior is moistened by serum, which may increase in quantity, and form encysted hydroceles of the cord.

5thly. I have many times seen the tunica vaginalis very much elongated, and extending upwards in front of the cord, into the inguinal canal, to become continuous with the depression of the peritoneum through the intervention of a solid cellular fasciculus.

In all these cases, the tunica vaginalis is completely separated from the peritoneum ; it may, however, preserve a communication with that membrane. The depression which the latter presents is then continuous with a canal which is either long and contracted at different points, or short and wide, and consists simply of the upper extremity of the tunica vaginalis. This canal allows the serum of the abdomen to flow into the tunica vaginalis, thus forming a hydrocele. It may also become the sac of a hernial protrusion." 26.

This concludes the anatomy of inguinal hernia. To recapitulate would be superfluous, and we shall content ourselves with directing the attention of our surgical readers, to the observations and researches of our author. There are many parts of anatomy, which, for practical purposes, may be superficially remembered. But the study and the recollection of those connected with the greater operations of surgery, or, indeed, with the less imposing manipulations of it, can never be too minute and circumstantial. It is the possession of this elaborate description of knowledge which distinguishes, in the hour of difficulty and of need, the scientific and accomplished, from the routine operator.

We proceed to the anatomy of the parts concerned in femoral hernia.

### Anatomy of the Parts concerned in Femoral Hernia.

M. Cloquet commences by a precise and correct description of the sinuous fossa comprised between the anterior superior spinous process of the ilium, and the spine of the pubes. Between those two points is extended the lower border of the aponeurosis of the external oblique, in other words, the ligament of Poupart, or the crural arch.* But the point of most consequence, because the most litigated, is the disposition of that expansion from the ligament of Poupart, usually known as the ligament of Gimbernat. M. Cloquet's account of this is valuable and perspicuous.

" In addition to the principal attachment of Poupart's ligament to the spine of the pubes, it also has an insertion into the crista of this bone by means of a fibrous triangular expansion, which is detached from its posterior part. This expansion has a direction almost horizontal in the upright posture of the body, so that it has an anterior border and rather superior, which is fixed to the crural arch; and a posterior inferior border, which is attached to the whole length of the crista of the pubes. Its base turned outwards corresponds with the iliac vessels; it is thin, rather concave, and is continuous with a fibrous lamina more or less strong, which I shall presently consider,—its summit is narrow and terminates at the spine of the pubes; this ligament, therefore, fills internally the triangular space between the pubes and the crural arch.

This expansion must not be considered as distinct from the crural arch; for, as the latter approaches the pubes, it increases in breadth, to be fixed to its spine and crista by means of this expansion, which appears to be reflected beneath the inferior column of the inguinal ring.

The ligament now described varies in dimensions; ordinarily its great diameter is from six to ten lines. Dr. Monro observes, that its strength is greater in the male than in the female, and to this he refers the comparatively rare occurrence of femoral hernia in the former; this observation, however, is not invariably correct: in some females I have found the ligament stronger and broader than in many males; in other instances, it has presented no differences in the

---

* It may be well to inform our provincial readers, that anatomical description has of late years become more minute and more methodical.

two sexes. In some subjects this expansion of the crural arch is entirely cellular; in others, it does not even exist; occasionally it possesses considerable strength. It is almost constantly perforated by one or many small apertures for the passage of lymphatic vessels, at the point where it is continuous with the anterior wall of the crural canal, as will be afterwards seen.

A careful dissection of this ligament, which I shall call Gimbernat's ligament, will show that it consists in most subjects of two distinct laminæ, easily separable above, but intimately connected below, to be inserted together into the crista of the pubes. Of these two laminæ, one is posterior and deep,—it is continuous with the fascia transversalis, and the tendon of the rectus abdominis; the other is anterior and superficial; and is continued into the inferior column of the ring. A correct knowledge of the structure of Gimbernat's ligament is obtained by dissecting it towards the inguinal canal." 30.

The disposition of the fascia iliaca, its connexion with the tendon of the psoas parvus, when that muscle really exists, and its continuity with the fascia transversalis, are matters which we must dispose of with this brief notice. But this we must observe, that the continuity of the fascia iliaca and fascia transversalis, where the iliacus internus muscle passes beneath the crural arch, occasions a fibrous cul-de-sac, which constitutes a powerful means of preventing the escape of the viscera of the abdomen beneath the outer part of the crural arch.

The fascia iliaca, in passing to the thigh, forms between the crural arch and psoas muscles a border, which limits externally the superior opening of the crural canal, whilst Gimbernat's ligament limits it internally.

M. Cloquet dwells at considerable length on the crural canal, the passage through which femoral hernia proceeds, and analogous in this respect to the inguinal canal, which bears the same relation to the hernia of that name. A knowledge, then of the anatomy of the crural canal is absolutely necessary for an accurate acquaintance with that of the femoral hernia. We shall follow M. Cloquet, for we cannot materially abridge his statements.

*Of the Crural Canal.*

"In dissecting the upper and anterior crural region, which might be called the inferior inguinal, we meet, in proceeding from without inwards, 1st, the skin; 2ndly, adipose cellular tissue, which, in some individuals, may form a layer of two inches in thickness; 3rdly, the fascia superficialis, of which I have already spoken; 4thly, beneath the latter, the fascia lata which in this region has two origins, each by a distinct layer. The two layers which constitute these origins are separated from each other; the one, anterior and superficial, is fixed to the inferior border of the crural arch, and passes in front of the femoral vessels; whilst the other, posterior and deep, glides under these vessels, to be attached to the pubes, covering the pectinalis muscle, and becoming continuous at the linea-ilio-pectinea with that portion of the fascia iliaca which descends in front of the psoas and iliacus muscles. These two layers, by their separation, form a fibrous canal, which I shall call the crural canal, giving passage to the femoral artery and vein, and containing also absorbent vessels and glands." 32.

M. Cloquet describes next the oval aperture in the fascia lata, through which the saphena major vein passes in for the purpose of joining the femoral. This aponeurotic aperture varies considerably as to its distance below Poupart's ligament, and also in its dimensions and form; in some instances it is separated from it by a considerable space, the extent of which is determined by the point where the vena saphena unites with the femoral vein. It is generally nearer to the crural arch in the female than in the male; its great diameter, which is vertical, is from six to ten lines in length; its small diameter is transverse, and measures about eight lines; its upper extremity, not very clearly defined, in some subjects, is separated from the crural arch by an interval of only three or four lines; in others, the distance amounts to an inch or an inch and a half. The inferior extremity is formed by an aponeurotic semi-lunar fold, strong and distinct, the concavity of which is directed upwards towards the crural arch, and is received into the angle formed by the junction of the vena saphena and femoral vein. This crescentic edge is continuous externally with that portion of the fascia lata which covers the outer part of the thigh, as also with the anterior layer of the fascia, which ascends to be attached to the crural arch. Internally, the crescentic edge is continuous with the posterior layer of the fascia lata, which, covering the pectinalis and adductor longus, is attached to the pubes; it is also continuous with the superficial portion of the same fascia which constitutes the anterior wall of the crural canal. We here distinguish clearly the separation of the fascia lata into two layers; one ascending obliquely inwards and towards the inferior border of the crural arch and the lower column of the external ring, and covering the femoral vessels, whilst the other takes a direction beneath them to be attached to the pubes.

" The superficial layer of the fascia lata crosses in some degree the direction of the deeper-seated one; it dips beneath the crural arch to be continued into Gimbernat's ligament.* When the thigh is extended, and rotated outwards, the superficial layer of the fascia lata, composing the anterior part of the crural canal, is in the most complete state of tension; at the same time, the crural arch is stretched, and drawn downwards without producing much effect upon Gimbernat's ligament. By the flexion and rotation inwards of the thigh the opposite effects will be produced." 34.

M. Cloquet considers it as proper to view the crural aperture as a true canal for the transmission of the femoral vessels, as it is to deem the channel through which the spermatic cord passes out of the abdomen, an inguinal canal. As such, then, M. Cloquet methodically describes it. He takes in succession its superior aperture, its walls, and its inferior aperture. We are unwilling to omit the exact description, and we cannot essentially abridge it; we shall therefore give it unmutilated and entire.

" The superior aperture of the crural canal is situated above the pubes; it is triangular, inclines downwards and forwards, and presents three borders and three angles. One of these borders is anterior and superior; this is the longest; and is formed by the crural arch. The two others, are, one posterior and internal, the other, posterior and external.

The posterior and internal border is the shortest; it corresponds with the

---

* " In some subjects the continuation of the superficial layer with Gimbernat's ligament, is entirely cellular; and the aperture of the vena saphena in these instances is of large size, and of an irregular form. The layer in question presents a large falciform edge, the upper extremity of which becomes contracted to be inserted beneath the inferior pillar of the ring: the lower extremity supports the vena saphena by its concavity."

upper edge of the pubes, and with the deep layer of the fascia lata, which is attached to that part, and is here remarkably thick.

The posterior external border is of an intermediate length, compared with the two preceding. It is formed by the expansion of the tendon of the psoas parvus, (pelvic aponeurosis), which descends beneath the crural arch, and accompanies the united psoas and iliacus muscles.

Of the three angles, the internal is formed by Gimbernat's ligament; the external, by the concave aponeurotic fold, which is in the opposite direction, between the crural arch and the psoas and iliacus. The posterior is not clearly defined, and corresponds with the linea ilio-pectinea.

Walls of the crural canal.—This canal extends between the opening just described, and that which gives passage to the vena saphena. Its length varies, and depends upon the height at which this vein opens into the femoral; it measures from six to fifteen lines. Its direction is nearly vertical; it is triangular, and wider above than below. In the female it is a little shorter, buf generally wider than in the male.

The crural canal presents three walls: the anterior extends from the crural arch to the upper part of the opening for the vena saphena, and is formed by the superficial layer of the fascia lata, which ascends in front of the femoral vessels; it is much stronger externally than on the inner side, where it is continuous with the deep layer of the same fascia, and with Gimbernat's ligament. It is covered by skin, subcutaneous cellular tissue, and fascia superficialis; it adheres closely to the latter at its lower part. In front of it also are absorbent glands, and the superficial vessels of the groin. It covers the femoral artery and vein, and usually sends between these vessels two fibro-cellular partitions, which form the sheath of the vessels, and are attached to the posterior external wall of the crural canal. On the inner side of the femoral vessels, and between the anterior and the posterior internal walls is the space, through which the sac of a femoral hernia escapes; this space, however, is closed superiorly towards the abdomen by a septum, presently to be described.

Of the two posterior walls of the crural canal, the internal is formed by the deep layer of the fascia lata; it is narrow, and covered a little externally by the femoral vein: in front, it is separated from the anterior wall by the space just mentioned. Small round apertures are sometimes found in it, for the passage of lymphatics. Internally, it is united with the anterior wall, and is also continuous with the fascia lata, covering the muscles on the inner part of the thigh.

The posterior external wall is slightly convex and narrow, and is formed by the aponeurotic expansion of the psoas parvus, which covers the psoas and iliacus muscles and crural nerve: the femoral vessels and lymphatic trunks rest upon it.

Inferior aperture.—The inferior aperture of the crural canal is formed by the opening in the fascia lata, which gives passage to the vena saphena. This aperture, already described in part, is oval, and not very clearly defined; it sends a fibro-cellular prolongation over the upper part of the vena saphena, and which is continuous with the fascia superficialis. Inferiorly, its edge is strong and well defined, and supports the angle formed by the union of the vena saphena and femoral vein. By its continuity with the fascia superficialis, which covers it, a sort of half-spiral turn is formed, which, however, is not equally manifest in all subjects. Besides the fascia superficialis, absorbent glands, varying in size and situation, are also placed in front of this aperture. It gives passage to lymphatics and to subcutaneous arteries and veins belonging to the genital organs, and to the integuments of the groin and abdomen. Below it, the fascia lata passes externally upon the sartorius muscle, to which it furnishes a sheath; internally, it extends upon the adductor longus." 37.

From the foregoing description it will appear, what is the fact, that different portions of the crural canal exhibit different axes. The axis of the superior aperture of the canal, resting on the pubes, is downwards and forwards—that of the canal itself is vertical—and that of the inferior aperture is again directly forwards, corresponding, in fact, with the axis of the aperture for the vena saphena. An acquaintance with these facts is indispensably required, in order to know the course of crural hernia, and the best means of applying the taxis.

The external iliac artery and vein, in their transit through the crural canal, rest upon its two posterior walls, but chiefly on its posterior external. They are confined by two laminæ stretched between the anterior and posterior walls of the canal; one placed between the artery and vein: the other on the inner side of the latter, and lost in the surrounding cellular tissue, and in the septum which closes the entrance of the crural canal. The second lamina separates the sac of a crural hernia from the crural vein.

We cannot refrain from inserting the results of our author's observations on the obturator artery. They establish a broad result, highly interesting to the scientific surgeon.

"The epigastric artery often arises by a trunk, which is common to it and to the obturator; the latter vessel, however, more frequently is furnished by the hypogastric itself, or one of its branches, and has then no connexion with the crural canal. When there exists a common trunk for these two vessels, they usually separate from each other on the outer side of the superior aperture of the crural canal; occasionally, however, on its inner side, but rarely below that opening. In the first case, the obturator artery takes a direction downwards and inwards towards the thyroid foramen, and is completely on the outer side of the superior aperture of the crural canal. In the second case, this artery descends almost vertically behind the superior aperture, its proximity to Gimbernat's ligament then depending upon the length of the common trunk. Lastly, in the third case, the common trunk passes into the crural canal, or perhaps takes its origin within it, and the two branches derived from it re-enter the abdomen; the obturator artery, more or less tortuous, ascends, and turns over the upper border of the pubes into the cavity of the pelvis, towards the thyroid foramen; the epigastric artery turning underneath the crural arch, then runs upwards and inwards towards the rectus muscle. These varieties in the origin of the obturator artery, determine its relations to the sac of a crural hernia."*

Some other varieties of the origin of the obturator are observed. We need not allude to them more in detail. The only remaining point which we shall notice is the septum crurale, a name applied to the cribriform fascia of Sir Astley Cooper.

"The superior orifice," says he, "of the crural canal is closed by a membranous septum, which tends to prevent the occurrence of femoral hernia, and opposes the entrance of the finger when we endeavour to introduce it from above

---

* I have studied with much care the relations which the epigastric, obturator, or their common trunks may have with regard to the crural canal; I have drawn and described the most interesting varieties in these arteries, both in the healthy state and in crural hernia; further, I have endeavoured to establish, in a given number of subjects, the proportion in which the obturator artery arises from the hypogastric, from the external iliac, or from the epigastric; with that view, I have examined these vessels in two hundred and fifty subjects, the half of which were males. The following were the results obtained :—

downwards, beneath the crural arch. This septum constitutes, in some subjects, a sort of fibro-cellular partition, of a very firm and resisting nature; in others, it is of a thin cellular structure, and easily yields to the pressure of the finger. I propose to give it the name of Septum Crurale. The following is its usual disposition:—it arises from the circumference of the superior aperture of the crural canal; it is tolerably thick; its fibres are generally transverse anteriorly, and towards the crural arch; internally it is derived from the cellular tissue, behind Gimbernat's ligament, and, perhaps, even from the concave edge of the latter, conjointly with the anterior boundary of the crural canal; externally it is confounded with the sheath of the femoral vessels, and with the cellular tissue which surrounds the epigastric artery. On the outer side of this artery we find also that the interval between the crural arch and the femoral vessels is filled with cellular membrane.

The upper surface of the Septum Crurale is towards the abdominal cavity; it is concave: the inferior, directed towards the crural canal, is convex: each surface is, however, sometimes level. This septum is always perforated by small apertures for the passage of lymphatics, and which are sometimes so numerous, that the superior part of the canal appears to be closed simply by a fibro-cellular net-work. One of these apertures, more considerable than the others, is central, and is sometimes occupied by an elongated absorbent gland; it is sufficiently large to admit the point of the little finger, which, when introduced, will be girt by it as by an elastic fibrous ring. Internally, another tolerably large foramen is also sometimes found, near to Gimbernat's ligament." 41.

Here we close the subject and the volume. The importance of the former

---

| | | | | |
|---|---|---|---|---|
| Obturator artery, arising from | 1st. Hypogastric on both side .... | in | 160 subjects, | 87 males, 73 females. |
| | 2nd. Epigastric on both sides .... | in | 56 subjects, | 21 males. 35 females. |
| | 3rd. Hypogastric on one side; from the epigastric on the other ................ | in | 28 subjects, | 15 males. 13 females. |
| | 4th. The femoral .............. | in | 6 subjects, | 2 males. 4 females. |
| | Total........ | | 250 subjects, | 125 males, 125 females. |

The following is the relative proportion of cases in which the obturator has or has not a relation with the hernial sac—placing on one side the cases of obturator arising from the epigastric, or directly from the femoral; and on the other, those arising from the hypogastric, we find

| | | | | |
|---|---|---|---|---|
| Obturator artery arising | From the hypogastric............ | in | 348 subjects, | 191 males, 157 females. |
| | From the epigastric or femoral.... | in | 152 subjects, | 58 males, 94 females. |
| | Total.......... | | 500 | |

From this calculation we find, 1st, that the cases in which the obturator takes its origin from the hypogastric are the most numerous; that their proportion, when compared with those in which it arises from the epigastric or femoral, is nearly as three to one. 2ndly, That the obturator appears to arise more frequently from the hypogastric in the male than in the female."

has induced us to depart from our usual custom, in directing so much attention to the latter; and, to the young and the old scientific anatomist and surgeon we earnestly recommend the study of both.

